# Association between Psychological Status and Condylar Bony Changes in Adults: A Retrospective Survey-Based Study

**DOI:** 10.3390/jcm11247497

**Published:** 2022-12-17

**Authors:** Chenghao Zhang, Ling Ji, Zhihe Zhao, Wen Liao

**Affiliations:** 1 State Key Laboratory of Oral Diseases & National Clinical Research Center for Oral Diseases, Department of Orthodontics, West China Hospital of Stomatology, Sichuan University, Chengdu 610041, China; 2 State Key Laboratory of Oral Diseases & National Clinical Research Center for Oral Diseases, West China Hospital of Stomatology, Sichuan University, Chengdu 610041, China

**Keywords:** psychological status, condyle, TMD, CBCT, orthodontic

## Abstract

Background: This article studies the association between psychological status and condylar bony changes in adults and assesses psychological questionnaires as an indicator of potential condylar bony changes. It is meaningful because condylar bony changes, a severe subtype of TMD and big concern in orthodontic treatment, would likely be ignored in patients with few TMD symptoms, in which case, even with potential psychological problems (depression, somatization and anxiety) being noticed, orthodontists may underestimate the possibility or severity of condylar bony changes and thus may not perform relevant examinations. Methods: A total of 195 adults (145 female and 50 male) who consulted orthodontists and had clinical records were included in this study. Initial CBCT images taken before orthodontic treatment were used for observing their condylar bony changes, and a comprehensive questionnaire conducted by each adult to evaluate psychological status was collected. Age, sex, TMD symptom history, scores on Patient Health Questionnaire-9 (PHQ-9), PHQ-15 and Generalized Anxiety Disorder-7 (GAD-7), and condylar bony changes of these adults were recorded. Odds ratios (OR) and 95% confidence intervals (CIs) for identifying the independent risk factors associated with condylar bony changes were calculated with univariate and multivariate logistic regression analysis. The kappa statistic was used to assess intraobserver reliability of CBCT analysis. Results: The scores of PHQ-15 (OR: 2.088, 95% CI: 1.061–4.108, *p* < 0.05) and GAD-7 (OR: 2.133, 95% CI: 1.082–4.204, *p* < 0.05) were correlated with the condylar bony changes on CBCT. Besides, the probability of having condylar bony changes was positively correlated with the number of psychological problems concomitantly present in an adult (OR: 1.440, 95% CI: 1.10–1.867, *p* < 0.01). The intraobserver agreement value for CBCT analysis was substantial (κ = 0.732). Conclusions: This study found that positive PHQ-15 (somatization) and GAD-7 (anxiety) scores were associated with condylar bony changes in adults. Moreover, the number of psychological problems concomitantly present in an adult was positively associated with the probability of having condylar bony changes.

## 1. Introduction

Temporomandibular disorders (TMDs) are a heterogenous group of disorders including pain-related musculoskeletal disorders and painless function disturbances [1]. TMD has become a serious and complex health problem, with the current prevalence being about 15–31% among adults, which is on the rise in recent decades [2,3,4]. A general consensus is that the etiology of TMDs is multifactorial and can be attributed to both physical and psychological factors [5]. Recent studies have established the association between psychological factors and TMD, evidencing that the first onset of TMDs is strongly associated with previous life events, perceived stress, and negative affect, while somatic symptoms, psychosocial stress, and affective distress also play important roles in the development of TMD [6].

However, the correlation between psychological status and condylar bony changes, one of the most severe subtypes of TMD, has not been sufficiently studied. To specify and prove the degree of their association is important for the reason that condylar bony changes, as a big risk in orthodontic treatment, would likely be ignored since it is not necessarily accompanied by pain [7,8,9]. For patients who report little pain or few TMD symptoms, even if they show positive scores on psychological questionnaires, dentists may underestimate the severity of their TMD or even not think the patients have TMD, and thus not consider necessary doing comprehensive TMD diagnosis or other types of examination [10,11,12]. Therefore, if we could zoom in to configure the correlation between psychological status and condylar bony changes, the potential patient’s mental situation would be elevated as a significant indicator for this big yet sometimes less visible risk.

There is a wealth of self-administered questionnaires that can be used to assess psychological status, among which the most widely used are the 9-item version of the Patient Health Questionnaire (PHQ-9), 15-item version of the Patient Health Questionnaire (PHQ-15), and 7-item version of the Generalized Anxiety Disorder (GAD-7) [13]. The PHQ-9 is a depression assessment module consisting of 9 items scored from 0 to 3. The thresholds of the PHQ-9 for mild, moderate, moderately severe and severe depression are 5, 10, 15, and 20 respectively [14]. Similarly, it is a somatization module and used to measure the severity of 15 kinds of physical symptoms. The GAD-7 is an assessment questionnaire for anxiety disorders [15,16]. The validity, sensitivity, and reliability of these three questionnaires for screening psychological status have already been proved [17,18,19]. As recommended, the PHQ-9, PHQ-15 and GAD-7 can be used to assist clinicians without specific training in mental health [20,21]. Although a questionnaire may not be used as a stand-alone diagnostic tool, it is thought to be an efficient method for screening patients before starting treatment.

For the examination of bone-related diseases, cone-beam computed tomography (CBCT) is now widely used in dentistry for its unique advantage in three-dimensional (3D) imaging [22]. Although in TMD diagnosis, CBCT may not be the most commonly used diagnostic imaging technique, as the information it provides about soft tissues is limited, it is still desirable as an efficient and accurate method for the examination of the osseous structure of TMJ in orthodontics and orthognathic surgery [23].

The aim of this study was to explore the association between the psychological status and condylar bony changes in adults by matching the survey assessment of mental health-related questionnaires and CBCT findings.

## 2. Materials and Methods

### 2.1. Sample Collection

This was a retrospective survey-based study. Potential adult patients who had had orthodontic consultations in the Department of Orthodontics, West China Hospital of Stomatology, Sichuan University (Chengdu, China) from June 2018 to June 2022 were filtered in this study. Before orthodontic treatment, they were asked to take a CBCT examination and complete a comprehensively questionnaire that contained their basic information, TMD symptom history, and assessment using the PHQ-9, PHQ-15 and GAD-7. Those adults were filtered manually and selected as research samples based on the following inclusion and exclusion criteria.

The inclusion criteria were: (1) older than 18 years; (2) CBCT images were taken within 1 week before or after completing the questionnaire; (3) the imaging field of CBCT should three-dimensionally cover the whole structures of bilateral mandibular condyles; (4) no history of craniofacial syndrome or systemic disease.

The exclusion criteria were: (1) not all items of the questionnaire were finished; (2) insufficient quality of CBCT images, (3) history of immune disease, severe bone defects, or orthognathic surgery.

All the CBCT images were taken with the same CBCT machine (3D Accuitomo, Morita Group, Osaka, Japan), which was set according to the manufacturers’ recommendations (140 ∗ 100 mm FOV, 85 kV, 4.0 mA, and 360° rotation). The voxel size was 125 μm. The CBCT data were stored in DICOM multifile format.

### 2.2. Assessment of Condyle Morphology with CBCT Images

**Figure 1:**
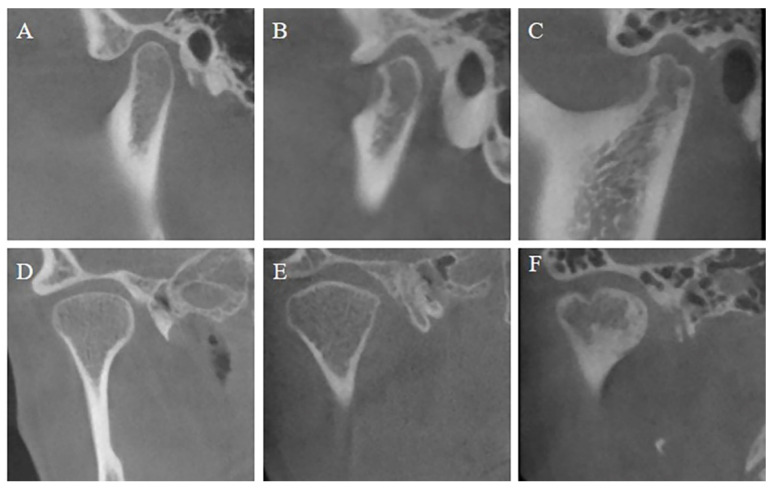
CBCT images of representative examples of condylar bony changes. (**A**) Sagittal view of normal condyle; (**B**) sagittal view of condyle with articular surface flattening; (**C**) sagittal view of condylar bony deformation; (**D**) coronal view of normal condyle; (**E**) coronal view of condyle with articular surface flattening; (**F**) coronal view of condylar bony deformation.

Before assessment, the DICOM data were imported into Dolphin software (Version 11.8; Dolphin Imaging & Management Solutions; Chatsworth, CA, USA) and the head positions were three-dimensionally reoriented with the Frankfort plane parallel to the ground. Then, the CBCT images without participant information were analyzed blindly under identical conditions. After a two-week interval, CBCT images were blindly analyzed by the same operator for the second time. The differences between the two analyses were marked out and discussed with an expert to confirm the results. According to previous research [10,24], the condyle morphology was divided into three subtypes: normal, articular surface flattening, and condylar bony deformation due to subcortical cyst, surface erosion, osteophyte, or generalized sclerosis (Figure 1).

Articular surface flattening in a young population indicates potential condylar resorption while the cortical margin is still intact [25]. It is noteworthy that TMJ flattening can be seen as a kind of indeterminate finding and may represent normal variation, due to aging or remodeling [26]. Hence, TMJ flattening in adults was considered a basically normal finding in this study. The degree of condylar bony changes was scored as 0 (normal condyles without bony deformation) and 1 (with condylar bony deformation) according to CBCT findings.

### 2.3. Analysis of Questionnaires

The questionnaires were analyzed on each item. Variables were classified into categorical and continuous types. The categorical variables in the questionnaire included sex and TMD symptom history and were then converted into continuous variables. Sex was scored as 0 (female) and 1 (male). TMD symptom history was scored as 0 (no symptoms ever) and 1 (symptom history including TMJ clicking and pain). The continuous variables included age and scores on the PHQ-9, PHQ-15, and GAD-7. According to the definition, the thresholds to be defined as a positive PHQ-9 score for depression, a positive PHQ-15 score for somatization, and a positive GAD-7 score for anxiety were 5. Further, the number of the psychological problems concomitantly present in a participant was defined in values (0–3), as shown in Table 1.

### 2.4. Data Analysis and Statistics

Based on the Kendall rule and events per variable (EPV) theory [27], the theoretical sample size of this study was as least 70. Statistical analysis was performed with SPSS software (version 22.0; IBM, Armonk, NY, USA). Quantitative data were described by means and standard deviations. Unpaired *t* tests were used to test the difference in age between the divided groups of participants (i.e., male and female; normal condyles and having condylar bony changes). The sociodemographic characteristics of the participants were analyzed using the *t* test or chi-squared test. The kappa statistic was used to assess intraobserver reliability for CBCT analysis. The relationships between degree of condylar bony changes and potential risk factors, including sex, age, TMD symptom history, PHQ-9, PHQ-15, and GAD-7 scores and number of psychological problems concomitantly present in a participant, were evaluated using univariate and multivariate logistic regression analysis. Multicollinearity among potential risk factors correlated with condylar bony changes were estimated. Odds ratios (ORs) and 95% confidence intervals (CIs) were calculated for risk factors. The threshold of statistical significance was set at 0.05.

## 3. Results

### 3.1. Baseline Characteristics

A total of 195 adults (145 females and 50 males) met the inclusion and exclusion criteria and were selected as research samples. Ages ranged from 18 to 54 years old; and the average age was 26.44 ± 6.68 years. No significant differences were found between females and males in terms of age.

According to the results of CBCT analysis and questionnaires, 128 participants (65.64%) were considered to have bilateral normal condyles (degree = 0), and 67 participants (34.36%) were considered to have condylar bony deformation (degree = 1). The intraobserver agreement value for CBCT analysis of condylar bony changes was substantial (κ = 0.732). No statistically significant differences in age were found between these two groups (Table 2).

**Figure 2:**
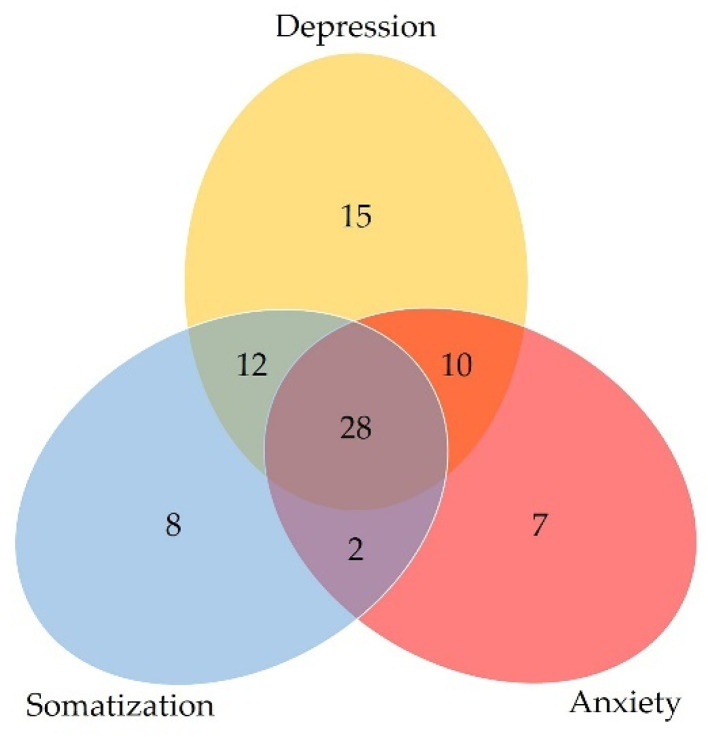
Venn diagram of the numbers of participants with psychological problems.

The PHQ-9, PHQ-15 and GAD-7 scores were analyzed, and we found that 42.05% of all the participants had at least one psychological problem. The numbers and percentages of participants with depression, somatization, and anxiety are listed in Table 3 and displayed in Figure 2.

Baseline characteristics of participants are shown in Table 4. Based on previous studies, age was classified into two categories: ≤30 years and >30 years [28,29]. As the results show, there were no statistically significant differences in age, sex or PHQ-9 score between participants with and without condylar bony changes, and there was a statistically significant difference in TMD symptom history between these two groups. Although there were no statistically significant differences in average PHQ-15 or GAD-7 scores between these two groups, the group with condylar bony changes had a higher proportion of participants with positive PHQ-15 and GAD-7 scores (*p* < 0.05). The proportion of participants with concomitant psychological problems was higher in the group with condylar bony changes than the group without condylar bony changes.

### 3.2. Univariate Logistic Regression Analysis of Potential Risk Factors

To identify the independent risk factors for condylar changes, univariate logistic regression analysis was firstly used and ORs were estimated. The results are shown in Table 5.

TMD symptom history (OR: 2.298, 95% CI: 1.132–4.665, *p* < 0.05), PHQ-15 score (OR: 2.126, 95% CI: 1.111–4.067, *p* < 0.05) and GAD-7 score (OR: 2.154, 95% CI: 1.105–4.197, *p* < 0.05) had statistically significant correlations with condylar bony changes. In addition, the number of psychological problems concomitantly present in a participant (OR: 1.440, 95% CI: 1.10–1.867, *p* < 0.01) was positively associated with the probability of having condylar bony changes.

### 3.3. Assessment of Potential Confounding Variables

Multicollinearity among potential risk factors correlated with condylar bony changes was estimated based on the data of all participants (Table 6). Condylar bony change was set as and dependent variable and input in the test as 0 (without condylar bony changes) and 1 (with condylar bony changes), and the potential risk factors were set as independent variables.

The variance inflation factors (VIFs) of age, sex and TMD symptom history were below 2, which indicated the absence of multicollinearity. The VIFs of the PHQ-9, PHQ-15 and GAD-7 scores were above 2 and below 5, which indicated moderate multicollinearity and were within acceptable level. The VIF of number of psychological problems concomitantly present in a participant was above 10, which indicated high multicollinearity and was considered problematic [30]; hence, this risk factor was not further analyzed.

Then, the above three risk factors with statistical significance (TMD symptom history, PHQ-15 and GAD-7 scores) were set as the dependent factors. Univariate logistic regression analysis was used again to figure out the potential confounding variables based on other risk factors (Table 7).

The results indicated that no confounding variable was statistically related to TMD symptom history. For PHQ-15 and GAD-7 scores, age, sex and TMD symptom history were not confounding variables. However, PHQ-9, PHQ-15 and GAD-7 scores were significantly correlated with each other. Therefore, these three psychological risk factors were seen as the confounding variables to each other and removed when assessing the association between condylar bony change and PHQ-15 and GAD-7 scores in the following multivariate logistic regression analysis.

### 3.4. Multivariate Logistic Regression Analysis

As we mentioned above, psychological risk factors were confounding variables to each other and removed in the multivariate statistics. Hence, multivariate logistic regression analysis was conducted twice to respectively estimate the ORs and significance of PHQ-15 score and GAD-7 scores associated with the condylar bony change, as shown in Table 8.

TMD symptom history (OR: 2.292–2.321, *p* < 0.05), PHQ-15 score (OR: 2.088, 95% CI: 1.061–4.108, *p* < 0.05) and GAD-7 score (OR: 2.133, 95% CI: 1.082–4.204, *p* < 0.05) showed statistically significant correlations with condylar bony changes.

## 4. Discussion

Our study aimed to explore the association between psychological status and condylar bony changes in adults. A comprehensive questionnaire was used to evaluate the psychological status of these adults when they consulted orthodontists. Condylar bony changes were examined using CBCT images. The results indicated that psychological status assessed with the PHQ-15 (OR: 2.088, 95% CI: 1.061–4.108, *p* < 0.05) and GAD-7 (OR: 2.133, 95% CI: 1.082–4.204, *p* < 0.05) was positively correlated with condylar bony changes, which means positive scores on the PHQ-15 and GAD-7 indicate a possibility of condylar changes. Moreover, the number of psychological problems concomitantly present in an adult is positively associated with the probability of condylar bony changes (OR: 2.645, 95% CI: 1.127–6.207, *p* < 0.05). Compared with previous research that proved a close association between psychological status and TMDs, this study innovatively combined a questionnaire survey and CBCT images and further revealed the correlation between psychological status and condylar bony changes in adults. A similar phenomenon between psychological problems and condylar bony changes was seen in some animal experiments that used classic animal models of anxiety and stress. To be specific, Jiao K et al. established a mouse model of chronic immobilization stress by placing mice in a ventilated 50 mL laboratory conical tube for 4 h daily for generating endogenous psychological stress, and found that such psychological stress promoted condylar subchondral bone loss and cartilage degradation [31]. Wu G et al. also revealed that using the well-established rat communication box model, the rats that experienced psychological stress via sensing the hair erection and screams of other rats would exhibit pathologic changes in condylar processes, while diazepam (a kind of anxiolytic agent) injection could reduce such condylar changes [32]. In a clinical study, a subgroup of patients who had significant abnormal findings of TMJ on magnetic resonance imaging (MRI), including disk replacement and effusion, had significantly high scores on psychological questionnaires [33].

Condylar bony changes also had an association with TMD symptom history (OR: 2.292–2.321, *p* < 0.05). This can be easily understood. TMD symptom history reported by patients themselves may reflect preexisting TMDs. Without removal of pathogenic factors or timely treatment, TMDs may result in TMJ osteoarthritis (OA) and even cause condylar bony deformation, which can be easily observed with CBCT images [8].

This study also indicated that PHQ-9, PHQ-15, and GAD-7 scores were confounding variables to each other (*p* < 0.001). As previous studies have shown, the relationship of depression, somatization and anxiety is a kind of comorbidity and can be called the “SAD” triad [13].

One limitation of this study was the unbalanced sex distribution. Fifty males were selected while the female number was one hundred and forty-five, and the percentages were 26.64% and 74.36%, respectively. This could be traced to the disparate willingness of adult males and females to undergo orthodontic treatment [34]. Another limitation is that the participants included in this study were adults who had once consulted orthodontists. While the prevalence of TMDs in adults was reported to be 15–31% approximately, the percentage of participants with condylar bony deformation in this study was higher (34.36%) according to CBCT analysis. Other studies also proved that although there were no disease-specific associations between TMDs and malocclusions, patients seeking orthodontic treatment have a significant incidence of TMDs when compared to a control population [35,36,37]. Therefore, we remind readers that more accurate results should be based on a more general population. Future studies are needed to validate the relationship between psychological status and condylar bony changes.

The results of this study could be meaningful for clinical practice in helping to promote an awareness of the relationship between psychological status and condylar bony changes in adults. On one hand, when patients report positive scores of psychological questionnaires or are diagnosed with psychological problems, orthodontists may consider the possibility of condylar bony changes. This is meaningful, as orthodontists may not tend to come to TMD diagnoses or conduct other examinations to confirm if patients have condylar bony changes; yet, condylar bony change has been an unignorable risk for future orthodontic treatment and the patients’ own health. In this regard, orthodontists and other dentists could more effectively use the PHQ-9, PHQ-15 and GAD-7 questionnaires, whose results could be considered indicators of condylar bony changes. In addition, when the questionnaire results indicate a patient has multiple psychological problems at the same time, an increased possibility of having condylar bony changes could be expected, in which case the orthodontists need to pay due attention. Confirmation is not difficult, as CBCT provides a convenient method to know whether the patients actually have condylar bony changes. Compared to MRI, which is admittedly the gold standard for TMJ imaging and can be used in assessing the status of the osseous as well as non-osseous structures of the TMJ, CBCT is a more efficient, convenient, and cost-saving choice that provides sufficiently accurate information about bony structures [38]. As the imaging field of CBCT images can cover the structures from bilateral condyles to mandible, CBCT could be taken only once for orthodontic examination, which could meanwhile be observed for examining condylar bony changes. On the other hand, when condylar bony changes are detected on CBCT, due attention should be paid to the patients’ psychological status, as it is not just an influencing factor in orthodontic treatment but also intimately related to the well-being of patients. Confirmation could be easily done with a wealth of questionnaires about psychological problems.

## 5. Conclusions

This study systematically investigated and found that psychological status assessed by psychological questionnaires (PHQ-15 and GAD-7) was associated with condylar bony changes in adults having orthodontic consultations. The number of the psychological problems (depression, somatization, and anxiety) concomitantly present in an adult is positively associated with the probability of having condylar bony changes. It indicated that screening for potential psychological problems using the PHQ-9, PHQ-15 and GAD-7 could be potentially useful for detecting condylar bony changes before orthodontic diagnosis and treatment of adults; vice versa, when condylar bony changes are detected, special attention should be given to the patients’ psychological condition throughout future treatment. Future studies are needed to validate the relationship between psychological status and condylar bony changes.

## Figures and Tables

**Table 1:** The value of the number of psychological problems concomitantly present in a participant.

Value	Number of Psychological Problems Concomitantly Present in a Participant
0	No psychological problems
1	Only one psychological problem, either depression, somatization or anxiety
2	Two of the three psychological problems
3	All three psychological problems

**Table 2:** Age characteristics of the participants.

Characteristic	Number of Participants	Age (Mean ± SD)	*p* Value
Sex			
Female	145	26.73 ± 6.68	0.295
Male	50	25.58 ± 6.69	
Degree of condylar bony changes			
0	128	26.62 ± 7.39	0.602
1	67	26.09 ± 5.10	

**Table 3:** Numbers of participants having psychological problems and percentage in all 195 participants.

Psychological Problem	Number	Percentage
Depression (PHQ-9)	65	33.33%
Somatization (PHQ-15)	50	25.64%
Anxiety (GAD-7)	47	24.10%
At least one of the above problems	82	42.05%

**Table 4:** Baseline characteristics of the participants and subgroup analysis.

Variables	Total (*n* = 195)	Without Condylar Bony Changes (*n* = 128)	With Condylar Bony Changes (*n* = 67)	*p* Value
Age	26.44 ± 6.68	26.62 ± 7.39	26.09 ± 5.10	0.602 ^a^
18–30	151 (77.44%)	97 (75.78%)	54 (80.60%)	0.686
>30	44 (22.56%)	31 (24.22%)	13 (19.40%)	
Sex				
Female	145 (74.36%)	94 (73.44%)	51 (76.12%)	0.593
Male	50 (25.64%)	34 (26.56%)	16 (23.88%)	
TMD symptom history				
0 (Without)	155 (79.49%)	108 (84.38%)	47 (70.15%)	0.019 *
1 (With)	40 (20.51%)	20 (15.62%)	20 (29.85%)	
PHQ-9 score	3.56 ± 4.12	3.24± 3.95	4.16 ± 4.39	0.138 ^a^
0–4	130 (66.67%)	89 (69.53%)	41 (61.19%)	0.243
≥5	65 (33.33%)	39 (30.47%)	26 (38.91%)	
PHQ-15 score	3.04 ± 3.65	2.85 ± 3.77	3.37 ± 3.39	0.351 ^a^
0–4	145 (74.36%)	100 (78.13%)	45 (67.16%)	0.021 *
≥5	50 (25.64%)	28 (21.87%)	22 (32.84%)	
GAD-7 score	2.53 ± 3.16	2.38 ± 3.28	2.82 ± 2.93	0.351 ^a^
0–4	148 (75.90%)	103 (80.47%)	45 (67.16%)	0.023 *
≥5	47 (24.10%)	25 (19.53%)	22 (32.84%)	
Number of psychological problems concomitantly present in a participant				
0	113 (57.95%)	83 (64.84%)	30 (44.77%)	0.001 **
1	30 (15.38%)	15 (11.72%)	15 (22.39%)	
2	24 (12.31%)	13 (10.16%)	11 (16.42%)	
3	28 (14.36%)	17 (13.28%)	11 (16.42%)	

All values are presented as the mean ± standard error or frequency (*n*, weighted %); *p*-values were obtained by means of the chi-squared test; ^a^ *p*-value was obtained by means of the *t*-test; * *p* < 0.05; ** *p* < 0.01.

**Table 5:** Univariate logistic regression analysis of potential risk factors for condylar bony changes.

Factors	OR	95% CI	*p* Value
Age			
18–30	1 (reference)	0.437–1.721	0.684
>30	0.867		
Sex			
Female	1 (reference)	0.556–2.805	0.591
Male	1.249		
TMD symptom history			
0 (No)	1 (reference)	1.132–4.665	0.021 *
1 (Yes)	2.298		
PHQ-9 score			
0–4	1 (reference)	0.779–2.687	0.242
≥5	1.447		
PHQ-15 score			
0–4	1 (reference)	1.111–4.067	0.023 *
≥5	2.126		
GAD-7 score			
0–4	1 (reference)	1.105–4.197	0.024 *
≥5	2.154		
Number of psychological problems concomitantly present in a participant			
0	1 (reference)		
1–3	1.440	1.110–1.867	0.006 **

OR, odds ratio; CI, confidence interval; * *p* < 0.05; ** *p* < 0.01.

**Table 6:** Multicollinearity test among potential risk factors correlated with condylar bony changes.

Risk Factors	Tolerance	Variance Inflation Factor (VIF)
Age	0.927	1.078
Sex	0.970	1.031
TMD symptom history	0.976	1.024
PHQ-9 score	0.246	4.072
PHQ-15 score	0.275	3.632
GAD-7 score	0.283	3.535
Number of psychological problems concomitantly present in a participant	0.071	14.169

**Table 7:** Univariate logistic regression analysis for estimating potential confounding variables.

TMD Symptom History			PHQ-15 Score			GAD-7 Score		
Factors	OR	*p* Value	Factors	OR	*p* Value	Factors	OR	*p* Value
Age	0.445	0.090	Age	0.284	0.070	Age	0.704	0.381
Sex	0.769	0.561	Sex	2.358	0.096	Sex	1.259	0.619
PHQ-9 score	1.098	0.802	PHQ-9 score	14.400	0.000 ***	PHQ-9 score	16.889	0.000 ***
PHQ-15 score	1.384	0.397	GAD-7 score	8.986	0.000 ***			
GAD-7 score	1.210	0.635						

OR, odds ratio; *** *p* < 0.001.

**Table 8:** Double multivariate logistic regression analysis to estimate the PHQ-15 score and GAD-7 score associated with the condylar bony change.

First Multivariate Logistic Regression Analysis				Second Multivariate Logistic Regression Analysis			
Factors	OR	95% CI	*p* Value	Factors	OR	95% CI	*p* Value
Age	1.141	0.553–2.358	0.721	Age	1.031	0.504–2.107	0.934
Sex	1.197	0.515–2.780	0.676	Sex	1.287	0.553–2.994	0.553
TMD symptom history	2.292	1.108–4.741	0.025 *	TMD symptom history	2.321	1.122–4.801	0.023 *
PHQ-15 score	2.088	1.061–4.108	0.033 *	GAD-7 score	2.133	1.082–4.204	0.029 *

OR, odds ratio; CI, confidence interval; * *p* < 0.05.

## Data Availability

Data can be provided upon reasonable request from the corresponding author.
